# CTHRC1 is associated with BRAF(V600E) mutation and correlates with prognosis, immune cell infiltration, and drug resistance in colon cancer, thyroid cancer, and melanoma

**DOI:** 10.17305/bb.2024.10397

**Published:** 2024-07-23

**Authors:** Rumeng Zhang, Zhihao Wang, Huan Wang, Lin Li, Lin Dong, Lin Ding, Qiushuang Li, Linyan Zhu, Tiantian Zhang, Yong Zhu, Keshuo Ding

**Affiliations:** 1 Department of Pharmacology, School of Basic Medical Sciences, Anhui Medical University, Hefei, Anhui, China; 2 Department of Pathology, School of Basic Medical Sciences, Anhui Medical University, Hefei, Anhui, China; 3 Department of Anesthesiology, The First Affiliated Hospital of Anhui Medical University, Hefei, Anhui, China; 4 Department of Pathophysiology, School of Basic Medical Sciences, Anhui Medical University, Hefei, China; 5 Department of Pathology, The First Affiliated Hospital of Anhui Medical University, Hefei, Anhui, China

**Keywords:** Collagen triple helix repeat containing 1 (CTHRC1), BRAF(V600E), colon cancer, thyroid cancer, melanoma

## Abstract

Colon cancer, thyroid cancer, and melanoma are common malignant tumors that seriously threaten human health globally. The B-Raf proto-oncogene, serine/threonine kinase (BRAF)(V600E) mutation is an important driver gene mutation in these cancer types. In this study, we identified that collagen triple helix repeat containing 1 (CTHRC1) expression was associated with the BRAF(V600E) mutation in colon cancer, thyroid cancer, and melanoma. Based on database analysis and clinical tissue studies, CTHRC1 was verified to correlate with poor prognosis and worse clinicopathological features in colon cancer and thyroid cancer patients, but not in patients with melanoma. Several signaling pathways, immune cell infiltration, and immunotherapy markers were associated with CTHRC1 expression. Additionally, a high level of CTHRC1 was correlated with decreased sensitivity to antitumor drugs (vemurafenib, PLX-4720, dabrafenib, and SB-590885) targeting the BRAF(V600E) mutation. This study provides evidence of a significant correlation between CTHRC1 and the BRAF(V600E) mutation, suggesting its potential utility as a diagnostic and prognostic biomarker in human colon cancer, thyroid cancer, and melanoma.

## Introduction

Colon cancer is the most common malignant gastrointestinal tumor and a leading cause of cancer-related death worldwide [1]. The five-year survival rate of patients with colon cancer is only 65% [2]. Thyroid cancer, especially papillary thyroid carcinoma [3], is one of the most common malignant tumors in the endocrine system, and its incidence rate has been steadily rising [4]. Melanoma, a cutaneous cancer caused by malignant melanocytes, ranks fifth in men and sixth in women among all cancers [5], with an increasing incidence rate in recent years [6]. Melanoma is a highly malignant tumor with a poor prognosis [7]. BRAF is a type of serine/threonine protein kinase that activates the MAP kinase/ERK signaling pathway [2, 8], which acts as a driver gene in colon cancer, thyroid cancer, and melanoma [9]. The main mutation type of BRAF is BRAF(V600E) [10]. The BRAF(V600E) mutation has been found in 8%–10% of metastatic colon cancer patients [11]; approximately half of papillary thyroid carcinomas and melanomas exhibit the BRAF(V600E) mutation [12]. However, BRAF mutations are rarely observed in other types of human cancers. BRAF and MEK inhibitors (BRAFi and MEKi) represent a breakthrough in the treatment of BRAF(V600E)-mutant cancers, greatly improving outcomes for cancer patients [13, 14]. In melanoma, immunotherapy targeting the BRAF(V600E) mutation represents a promising treatment option, especially for melanoma patients with traditional drug resistance [14, 15]. Besides traditional treatments, including surgery, radioactive iodine, and TSH suppression, BRAF(V600E)-targeted therapy is another option for thyroid cancer patients with a poor prognosis [16, 17]. However, primary and acquired drug resistance seriously impair the effectiveness of BRAF(V600E)-targeted therapy [18]. Therefore, it is crucial to study the molecular mechanisms involved in the BRAF(V600E) mutation in human colon cancer, thyroid cancer, and melanoma.

Collagen triple helix repeat containing 1 (CTHRC1), located on human chromosome 8q22.3, encodes an extracellular matrix glycoprotein with a molecular weight of 28 kDa [19, 20]. CTHRC1 has been reported to play critical roles in various human cancers. Overexpression of CTHRC1 increased migration and invasion of ovarian cancer cells by activating the EGFR/ERK1/2/AKT signaling pathway [21]. Elevated CTHRC1 was demonstrated to be an independent factor of a worse prognosis in gastric cancer [22, 23]. CTHRC1 promoted cell proliferation and invasion through the PI3K/Akt signaling pathway and was identified as a biomarker for prognosis prediction in bladder cancer [24, 25]. In colon cancer, CTHRC1 was shown to promote proliferation, migration, and invasion via activation of the Wnt/PCP pathway [26]. In papillary thyroid carcinoma, CTHRC1 was demonstrated to be correlated with tumor occurrence and malignant transformation, and it may play a crucial role in regulating EMT [27]. CTHRC1 has been reported to be overexpressed in melanoma cells, leading to melanoma metastasis [28, 29]. However, systematic prognostic analysis of CTHRC1 and its relation to the BRAF(V600E) mutation in human colon cancer, thyroid cancer, and melanoma still needs further investigation.

In this study, through database analysis and clinical tissue studies, we demonstrated that the expression of CTHRC1 was significantly associated with the BRAF(V600E) mutation in colon cancer, thyroid cancer, and melanoma. CTHRC1 expression was found to be negatively correlated with the overall survival (OS) rate in colon cancer and thyroid cancer patients and positively correlated with the OS rate in melanoma patients. Clinicopathological features, including tumor infiltration depth/clinical stage, and tumor size/lymph node metastasis/clinical stage, were positively correlated with CTHRC1 expression levels in colon cancer and thyroid cancer, respectively. Several signaling pathways, immune cell infiltration, and immunotherapy markers were correlated with CTHRC1 expression in colon cancer and thyroid cancer, though not as significantly in melanoma. In clinical tissue studies, colon cancer and thyroid cancer patients with the BRAF(V600E) mutation showed elevated CTHRC1 expression and immune cell infiltration. Moreover, high CTHRC1 expression levels were correlated with decreased sensitivity to BRAF(V600E) mutation-targeted drugs. Therefore, we provided evidence that CTHRC1 is associated with the BRAF(V600E) mutation and could be used as a diagnostic and prognostic biomarker in human colon cancer, thyroid cancer, and melanoma.

## Materials and methods

### Data collection based on TCGA database

Transcriptomic RNA-seq data of colon cancer, thyroid cancer, and melanoma samples, along with corresponding clinical characteristics, survival data, and somatic mutations, were collected from the TCGA database. Cases with missing or flawed information were excluded. Therefore, 454 colon cancer samples, 476 thyroid cancer samples, and 423 melanoma samples were included in this study.

### Correlation analysis of gene expression with BRAF(V600E) mutation

Correlation analysis of gene expression with the BRAF(V600E) mutation was carried out as previously described, using the limma (Version 3.52.3; http://www.bioconductor.org/ packages/release/bioc/html/limma.html) package in R software, the ggplot2 R package (Version 3.3.6; https://cran.r-project.org/web/packages/ggplot2), and the TIMER database-TIMER2.0 (http://timer.cistrome.org/) [30, 31]. For differential gene expression analysis in BRAF(V600E) mutant samples compared to wild-type samples, a log fold change (logFC) > 0.5 was considered statistically significant.

### Clinical samples

In this study, 50 colon cancer, 50 thyroid cancer, and 50 melanoma paraffin-embedded tissue samples were collected from the Department of Pathology, First Affiliated Hospital of Anhui Medical University (Hefei, Anhui, China). Among the 50 colon cancer tissues, 15 of them were BRAF(V600E) mutants and 35 of them were wild-type (WT); among the 50 thyroid cancer tissues, 30 of them were BRAF(V600E) mutants, and 20 of them were wild-type; and among the 50 melanoma tissues, 20 of them were BRAF(V600E) mutants, and 30 of them were wild-type. These tissue samples were obtained from patients who underwent surgical resection between 2016 and 2021. The clinicopathological parameters (including age, gender, tumor size, tumor infiltration depth, lymph node metastasis, distant metastasis, and clinical stage) of these patients were also collected. This work was performed in accordance with the World Medical Association’s Code of Ethics (Declaration of Helsinki). It was approved by the Institutional Review Board of Anhui Medical University, and informed consent was obtained from all patients.

### Quantitative real-time PCR (qRT-PCR)

Total RNA was isolated from paraffin-embedded tissues using an RNA-isolation kit (Thermo Fisher Scientific, USA). The mRNA levels of CTHRC1 were examined by qRT-PCR using SYBR green Master MIX (Applied Biosystem), as previously described [32, 33]. GAPDH was used as an endogenous control. The primer sequences were as follows: CTHRC1: 5′- TCATCGCACTTCTTCTGTGGA -3′ (forward) and 5′ GCCAACCCAGATAGCAACATC -3′ (reverse) [34]; GAPDH: 5′- TGGCCATTATAGGACCGAGACTT -3′ (forward) and 5′- CACCCTGTTGCTGTAGCCAAA -3′ (reverse).

### OS rate and ROC curve analysis

OS rates of colon cancer, thyroid cancer, and melanoma patients were derived from the TCGA-Clinical Data Resource (CDR). Kaplan–Meier curves were analyzed using the “survival” packages in R. The optimal cut point for CTHRC1 was determined using the R package “survminer,” based on the time of death of patients with colon cancer, thyroid cancer, and melanoma.

The area under the curve (AUC) of the ROC curve was calculated and plotted to evaluate the diagnostic effect of CTHRC1 in colon cancer, thyroid cancer, and melanoma, respectively.

### COX regression analysis

We used R version 4.1.2 software and the survival and survminer packages for COX regression analysis. Univariate COX regression analysis was performed to evaluate independent prognostic factors.

### Protein–protein interaction (PPI) and molecular pathway enrichment analysis

The STRING database was used to construct the PPI network of CTHRC1. Gene Ontology (GO) and Kyoto Encyclopedia of Genes and Genomes (KEGG) enrichment analyses were used to reveal the molecular pathways related to CTHRC1 in colon cancer, thyroid cancer, and melanoma, respectively.

### Correlation analysis of CTHRC1 expression with immune cells infiltrating

We collected data and analyzed the correlation of CTHRC1 expression with immune cell infiltration based on The TIMER database (TIMER2.0 (cistrome.org)). R version 4.1.2 software, along with the ggplot2, tidyverse (version: 1.3.2), and reshape2 (version: 1.4.4) packages, was used as appropriate [35].

### Immunohistochemistry

Immunohistochemistry (IHC) was performed to examine the protein levels of CTHRC1, Ki-67, and immune cell markers CD4, CD8, CD68, and CD69, essentially as described in previous studies, using the UltraSensitive-SP kit (Maixin-Bio, Fuzhou, China) [32]. CTHRC1 rabbit polyclonal antibody (Proteintech Group, Inc., Chicago, IL, USA, 1:100), Ki-67, CD4, CD8, CD68 mouse monoclonal antibodies (Maixin-Bio, Fuzhou, China, 1:1), and CD69 rabbit monoclonal antibody (Abcam, Cambridge, UK, 1:500) were used. Stained sections were evaluated independently by two senior pathologists using an Olympus microscope (Olympus America, Inc., Melville, NY, USA).

### Correlation analysis of CTHRC1 expression with drug sensitivity

Gene expression and drug susceptibility data for the same cancer samples were collected from the CellMiner database [36]. Data out of Clinical laboratory validation and FDA standard certification were excluded. Pearson correlation tests were performed to analyze the correlation between CTHRC1 expression and respective drug sensitivity.

### Ethical statement

The studies involving human participants were reviewed and approved by the ethics committee of Anhui Medical University. The patients/participants provided their informed consent to participate in this study.

### Statistical analysis

We performed statistical analysis using SPSS 20.0 and R version 4.1.2 software. Proportional risk hypothesis testing and fitted survival regressions were conducted in the survival analysis using the survival package, and the results were visualized using the survminer package, as well as the ggplot2 package. AUC analysis was conducted to evaluate the prognostic diagnostic performance of CTHRC1 for COAD, THCA, and SKCM. The Kolmogorov–Smirnov test was used to test the normality of the distribution. *T*-tests were performed for data that fit a normal distribution, while Mann–Whitney *U*-tests were used for data that did not fit a normal distribution or had a small amount of data (sample size less than 30). We used univariate Cox regression analysis, in which we classified a variety of continuous variables based on pathological examination of gross specimens after surgery to determine the TNM stage of the patient’s tumor and the clinical approach to tumor staging. For example, colon cancer and melanoma are most prevalent between the ages of 50 and 55, and thyroid cancer is more common in individuals over the age of 45. In pathology, the depth of infiltration for colon cancer stages T1–T2 indicates that the tumor is confined to the mucosal and basal layers, while T3–T4 indicates that the tumor invades to the plasma membrane and subplasma membrane. T-staging for thyroid cancer focuses on the volume of the tumor, with T1–T2 indicating a tumor volume of less than 4 cm confined to the thyroid gland, and T3–T4 staging indicating a tumor volume greater than 4 cm, accompanied by invasion of external tissues. T1–T2, for melanoma, indicates a tumor diameter of less than 5 cm without metastasis, while T3–T4 indicates a tumor diameter greater than 2 cm with metastasis. Clinical staging was based on TNM staging, with grades I–II indicating that the lesion was confined to the primary site without metastasis, and grades III–IV indicating the presence of lymph node or distant metastasis. We used the minimum cutoff value method of the R package “survminer” to classify high and low CTHRC1 expression. The Pearson chi-square test was used to analyze the differences in clinicopathological parameters between the high and low CTHRC1 expression groups. *P* < 0.05 was considered to be statistically significant (^*^P < 0.05, ^**^P < 0.01, ^***^P < 0.001).

**Figure 1.**
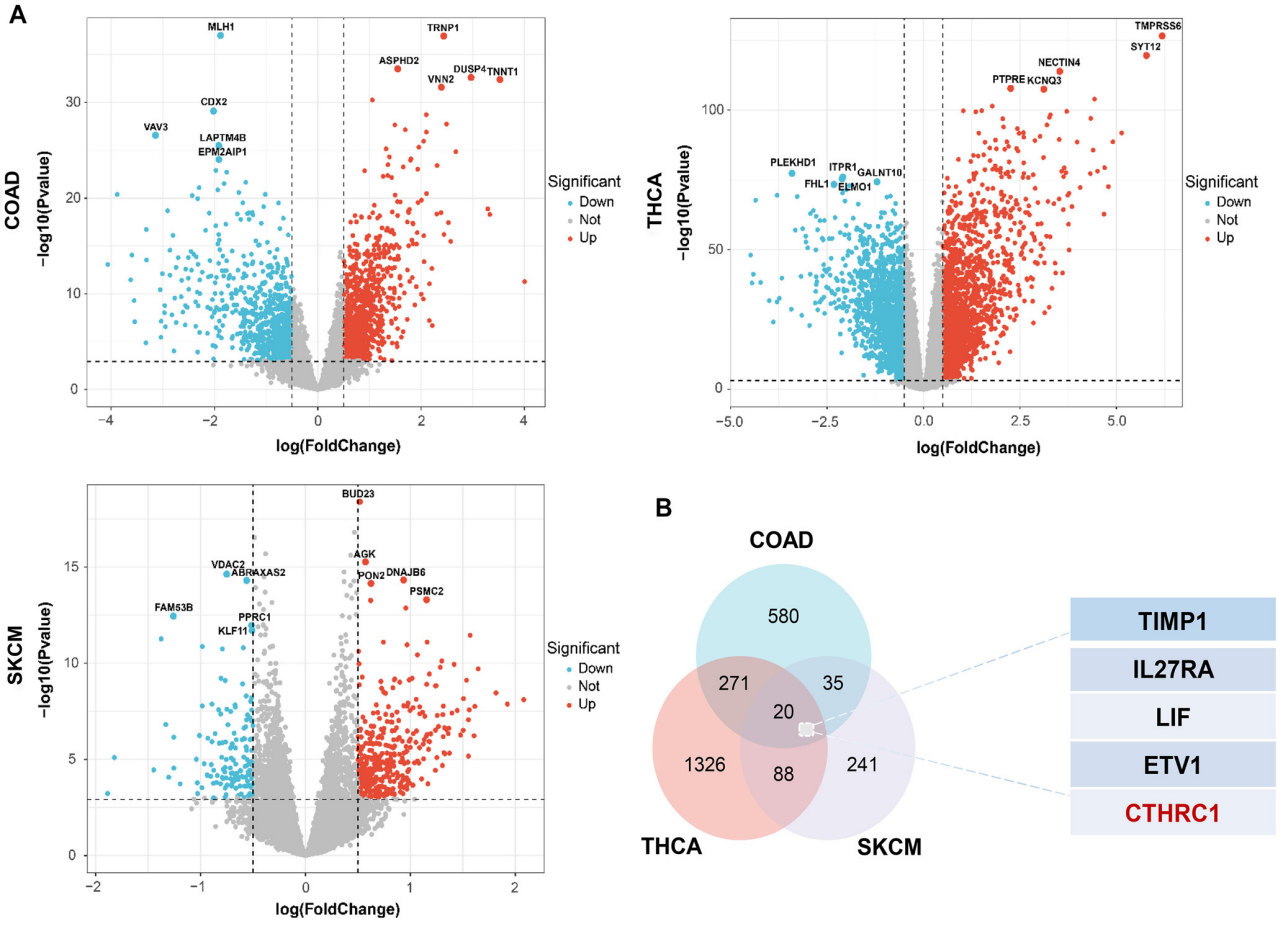
**Correlation analysis of BRAF(V600E) mutation with gene expression in colon cancer, thyroid cancer, and melanoma based on the TCGA database.** (A) Volcano plots showed genes that are downregulated or upregulated in BRAF(V600E) mutant tissues compared with wild-type tissues of colon cancer, thyroid cancer, and melanoma, respectively (COAD, colon cancer, THCA, thyroid cancer, SKCM, melanoma); (B) Intersection analysis of the genes up expressed in BRAF(V600E) mutant colon cancer, thyroid cancer, and melanoma tissues, with 5 potential oncogenes identified for further study.

**Figure 2.**
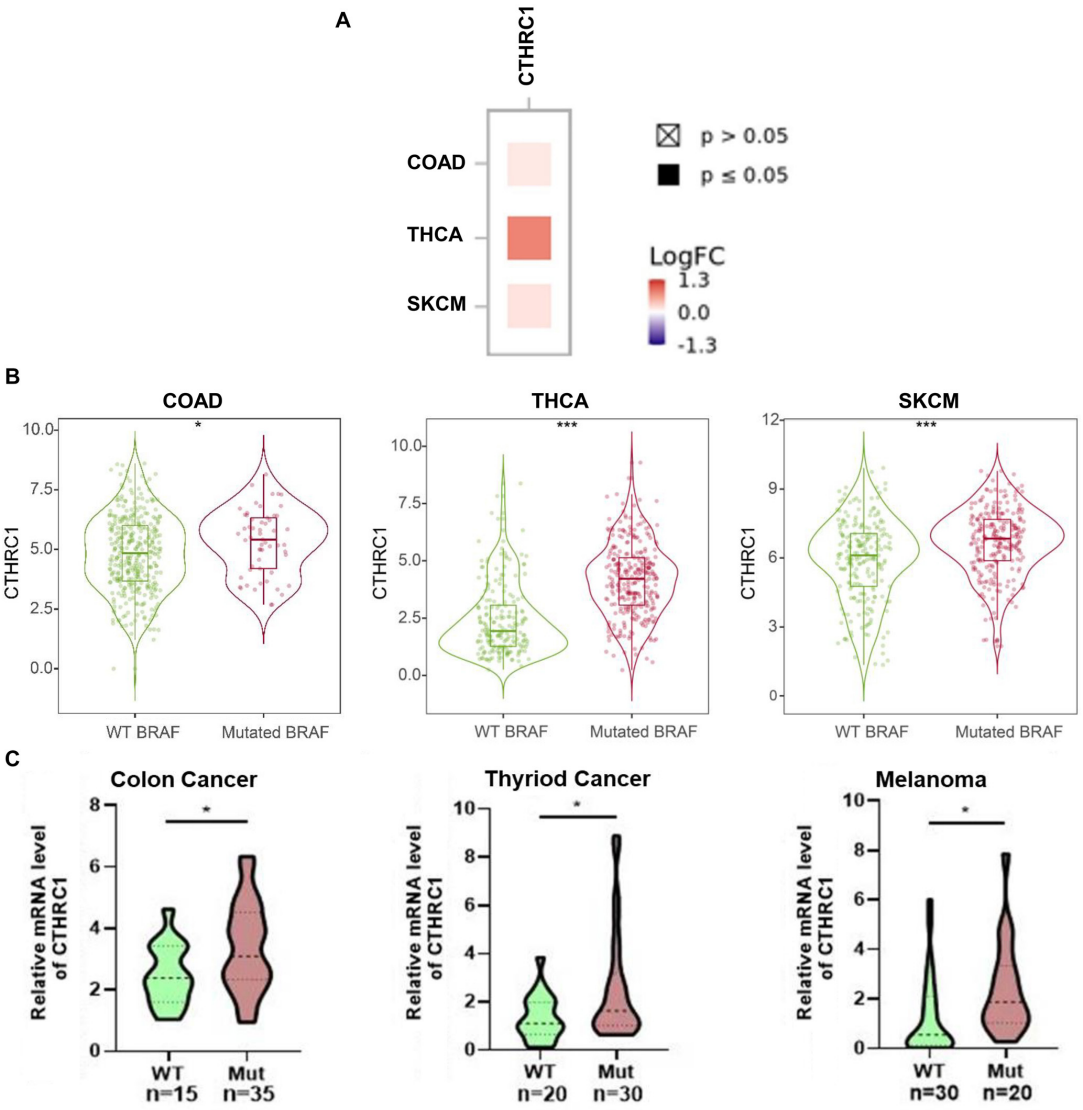
**CTHRC1 was highly expressed in BRAF(V600E) mutant colon cancer, thyroid cancer, and melanoma tissues.** (A) Correlation of CTHRC1 expression and BRAF(V600E) mutation in colon cancer, thyroid cancer, and melanoma tissues based on the TCGA database; (B) CTHRC1 mRNA expression in BRAF(V600E) mutant and wild-type colon cancer, thyroid cancer, and melanoma tissues based on the TCGA database; (C) CTHRC1 mRNA expression in BRAF(V600E) mutant and wild-type colon cancer, thyroid cancer, and melanoma clinical tissues collected from the Department of Pathology, First Affiliated Hospital of Anhui Medical University, as examined by qRT-PCR. *P < 0.05; ***P < 0.001. CTHRC1: Collagen triple helix repeat containing 1; qRT-PCR: Quantitative real-time PCR.

## Results

### Elevated CTHRC1 was associated with BRAF(V600E) mutation in colon cancer, thyroid cancer, and melanoma patients

BRAF(V600E) is a common gene mutation and is important in the prognosis and treatment options for human colon cancer, thyroid cancer, and melanoma. To explore the correlation between BRAF(V600E) and differential gene expression in colon cancer, thyroid cancer, and melanoma, we analyzed gene expression in the BRAF(V600E) mutant group and the wild-type BRAF group of colon cancer, thyroid cancer, and melanoma patients, based on the TCGA database. As shown in Figure 1A, many dysregulated genes were observed in the BRAF(V600E) mutant group compared to the wild-type BRAF group for colon cancer, thyroid cancer, and melanoma, respectively. By overlapping the elevated gene lists in BRAF(V600E) mutant colon cancer, thyroid cancer, and melanoma, 20 genes were screened out to be overexpressed in the BRAF(V600E) mutant group across all three tumor types (Figure 1B). Among these 20 genes, TIMP1, IL27RA, LIF, ETV1, and CTHRC1 were the top five most significantly correlated with the BRAF(V600E) mutation in human colon cancer, thyroid cancer, and melanoma; and only CTHRC1 was identified to be statistically significantly associated with the prognosis of all three tumor types (data will be shown later) (Figure 1B). Furthermore, the correlation between CTHRC1 expression and the BRAF(V600E) mutation in colon cancer, thyroid cancer, and melanoma was analyzed. As shown in Figure 2A, CTHRC1 expression and the BRAF(V600E) mutation were positively correlated in these three types of tumors, with correlation coefficients of 0.139 in colon cancer (COAD, *P* < 0.05), 0.794 in thyroid cancer (THCA, *P* < 0.05), and 0.179 in melanoma (SKCM, *P* < 0.05), respectively. Figure 2B shows that the expression levels of CTHRC1 were significantly higher in the BRAF(V600E) mutant group compared to the wild-type BRAF group in colon cancer, thyroid cancer, and melanoma, based on the public database. Moreover, we collected clinical tissue samples and examined the expression levels of CTHRC1 in colon cancer, thyroid cancer, and melanoma with/without the BRAF(V600E) mutation using qRT-PCR. As shown in Figure 2C, the RNA levels of CTHRC1 were consistently higher in tumor tissues with the BRAF(V600E) mutation compared to those without the mutation in colon cancer, thyroid cancer, and melanoma. Therefore, our data suggest that elevated CTHRC1 expression is positively associated with the BRAF(V600E) mutation in colon cancer, thyroid cancer, and melanoma patients.

### Association of CTHRC1 expression with prognosis in colon cancer, thyroid cancer, and melanoma patients

**Figure 3.**
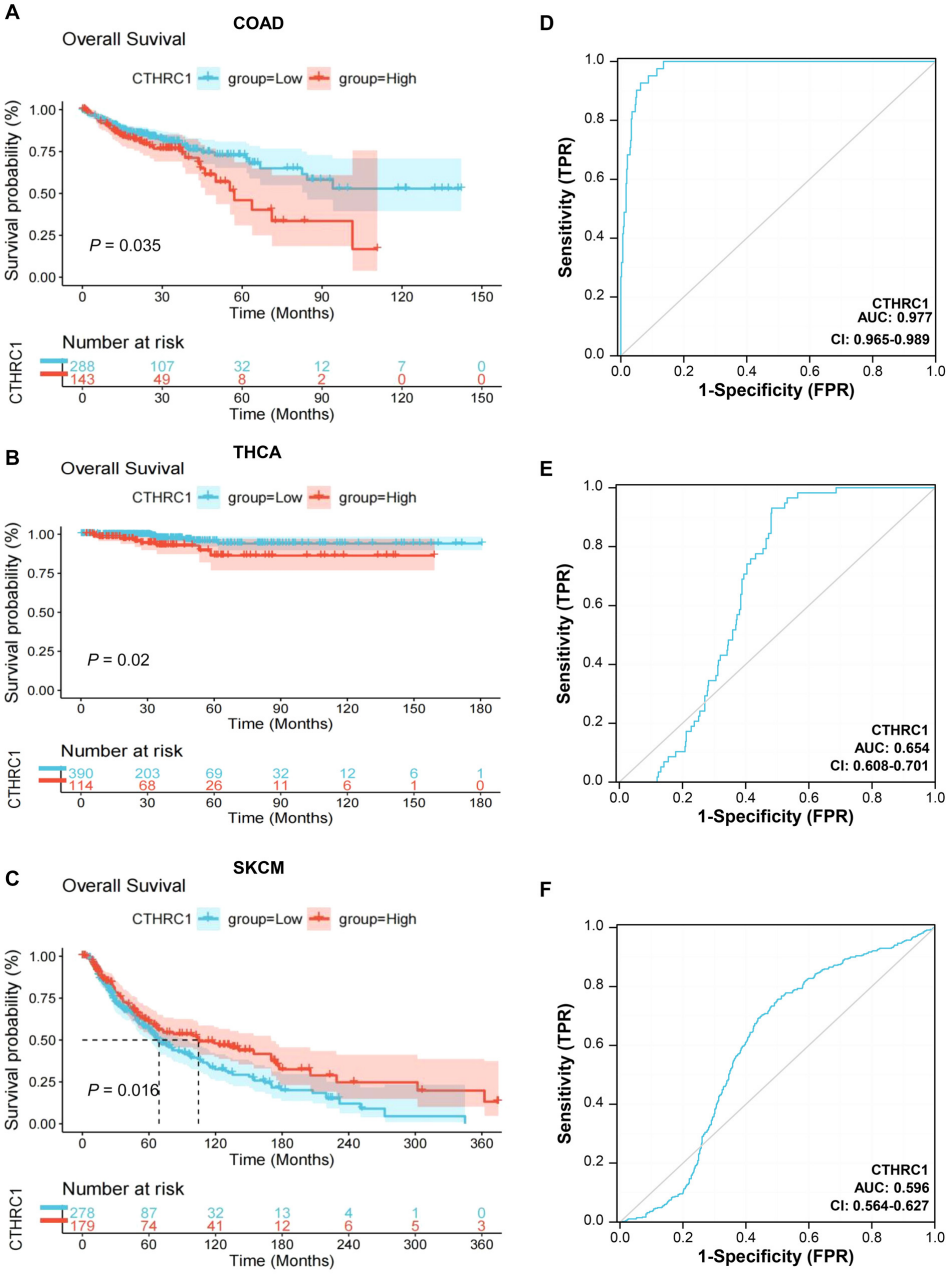
**OS rate and ROC curve analyses of CTHRC1 in colon cancer, thyroid cancer, and melanoma based on the TCGA database.** (A–C) Patient OS rates in the CTHRC1 high and low expression groups were analyzed using Kaplan–Meier analysis in colon cancer, thyroid cancer, and melanoma respectively; (D–F) The receiver operating characteristic (ROC) curves of CTHRC1 for the diagnosis of colon cancer, thyroid cancer, and melanoma respectively. CTHRC1: Collagen triple helix repeat containing 1; OS: Overall survival.

To further investigate, the correlation between CTHRC1 expression and survival rates in colon cancer, thyroid cancer, and melanoma patients was analyzed using Kaplan–Meier curves based on the TCGA database. As shown in Figure 3A, the OS rates of colon cancer patients with high CTHRC1 expression were significantly lower than those of colon cancer patients with low CTHRC1 expression (*P* ═ 0.035); similarly, a consistent trend was observed in thyroid cancer patients (*P* ═ 0.020) (Figure 3B). However, the OS rates of melanoma patients with high CTHRC1 expression were significantly higher than those of melanoma patients with low CTHRC1 expression (*P* ═ 0.016) (Figure 3C).

Moreover, we performed univariate COX regression analysis between prognostic risk factors and OS rates in colon cancer, thyroid cancer, and melanoma patients, respectively. As shown in Table 1, in colon cancer patients, significant correlations were observed between OS rates and risk factors, including depth of tumor infiltration (HR ═ 3.23) (*P* ═ 0.006), lymph node metastasis (HR ═ 2.73) (*P* < 0.001), distant metastasis (HR ═ 3.4) (*P* < 0.001), clinical stage (HR ═ 3.04) (*P* < 0.001), and CTHRC1 expression level (low and high) (HR ═ 1.56) (*P* ═ 0.036). In thyroid cancer patients, significant correlations were found between OS rates and risk factors, including tumor size (HR ═ 3.07) (*P* ═ 0.038), clinical stage (HR ═ 7.1) (*P* < 0.001), and CTHRC1 expression level (low and high) (HR ═ 3.03) (*P* ═ 0.027) (Table 2). In melanoma patients, significant correlations were found between OS rates and risk factors including age (HR ═ 1.74) (*P* < 0.001), tumor size (HR ═ 1.96) (*P* < 0.001), lymph node metastasis (HR ═ 1.82) (*P* < 0.001), distant metastasis (HR ═ 1.88) (*P* ═ 0.043), clinical stage (HR ═ 1.73) (*P* < 0.001), and CTHRC1 expression level (low and high) (HR ═ 0.71) (*P* ═ 0.017) (Table 3).

**Table 1:** COX regression analysis between prognostic risk factors and OS of colon cancer patients

**Characteristics**	**Total (*n*)**	**Univariate analysis**	
		**Hazard ratio (95% CI)**	*P*
Age			0.275
≤ 55	94	Reference	
> 55	358	1.34 (0.79--2.27)	
Gender			0.675
Male	239	Reference	
Female	213	1.09 (0.73--1.64)	
Tumor infiltration depth			**0.006**
≤ T2	89	Reference	
T3-T4	363	3.23 (1.41--7.4)	
Lymph node metastasis			**<0.001**
No	267	Reference	
Yes	185	2.73 (1.8--4.15)	
Distant metastasis			**<0.001**
No	331	Reference	
Yes	114	3.4 (2.23--5.19)	
Stage			**<0.001**
I–II	252	Reference	
III–IV	189	3.04 (1.96--4.73)	
CTHRC1			**0.036**
Low	305	Reference	
High	149	1.56 (1.03--2.36)	

CI: Confidence interval; CTHRC1: Collagen triple helix repeat containing 1; OS: Overall survival.

**Table 2:** COX regression analysis between prognostic risk factors and OS of thyroid cancer patients

**Characteristics**	**Total (*n*)**	**Univariate analysis**	
		**Hazard ratio (95% CI)**	***P***
Age			0.997
≤ 45	236	Reference	
> 45	266	737240933.9 (0-Inf)	
Gender			0.22
Male	138	Reference	
Female	367	1.89 (0.68--5.22)	
Tumor size (cm)			**0.038**
< 4	307	Reference	
≥ 4	196	3.07 (1.06--8.84)	
Lymph node metastasis			0.516
No	227	Reference	
Yes	229	1.45 (0.47--4.44)	
Distant metastasis			0.898
No	286	Reference	
Yes	218	0.94 (0.35--2.53)	
Stage			**<0.001**
I–II	83	Reference	
III–IV	167	7.1 (2.28--22.07)	
CTHRC1			**0.027**
Low	391	Reference	
High	114	3.03 (1.14--8.1)	

CI: Confidence interval; CTHRC1: Collagen triple helix repeat containing 1; OS: Overall survival.

**Table 3:** COX regression analysis between prognostic risk factors and OS of melanoma patients

**Characteristics**	**Total (*n*)**	**Univariate analysis**	
		**Hazard ratio (95% CI)**	*P*
Age			**<0.001**
≤ 55	116	Reference	
> 55	266	1.74 (1.32--2.3)	
Gender			0.354
Male	292	Reference	
Female	179	1.14 (0.86--1.51)	
Tumor size (cm)			**<0.001**
< 2	151	Reference	
≥ 2	244	1.96 (1.45--2.65)	
Lymph node metastasis			**<0.001**
No	235	Reference	
Yes	179	1.82 (1.36--2.43)	
Distant metastasis			**0.043**
No	418	Reference	
Yes	25	1.88 (1.02--3.46)	
Stage			**<0.001**
I–II	238	Reference	
III–IV	195	1.73 (1.3--2.31)	
CTHRC1			**0.017**
Low	286	Reference	
High	185	0.71 (0.54--0.94)	

CI: Confidence interval; CTHRC1: Collagen triple helix repeat containing 1; OS: Overall survival.

**Table 4:** Association of CTHRC1 expression with clinicopathological parameters in colon cancer patients based on TCGA database

**Characteristics**	**Low expression of CTHRC1 (%)**	**High expression of CTHRC1 (%)**	***χ^2^***	***P***
*n*	305	149		
Age			0.246	0.620
≤ 55	61 (20.1)	33 (22.1)		
> 55	242 (79.9)	116 (77.9)		
Gender			0.025	0.875
Male	161 (53.1)	78 (52.3)		
Female	142 (46.9)	71 (47.7)		
Tumor infiltration depth			5.521	**0.019**
≤ T2	69 (22.8)	20 (13.4)		
T3-T4	234 (77.2)	129 (86.6)		
Lymph node metastasis			2.660	0.103
No	187 (61.7)	80 (53.7)		
Yes	116 (38.3)	69 (46.3)		
Distant metastasis			0.069	0.757
No	223 (74.8)	108 (73.5)		
Yes	75 (25.2)	39 (26.5)		
Stage			4.221	**0.040**
I	59 (19.7)	16 (11.7)		
II-IV	241 (80.3)	121 (88.3)		

CTHRC1: Collagen triple helix repeat containing 1.

**Table 5:** Association of CTHRC1 expression with clinicopathological parameters in thyroid cancer patients based on TCGA database

**Characteristics**	**Low expression of CTHRC1 (%)**	**High expression of CTHRC1 (%)**	***χ^2^***	***P***
*n*	391	114		
Age			3.642	0.056
≤ 45	194 (49.6)	45 (39.5)		
> 45	197 (50.4)	69 (60.5)		
Gender			3.513	0.061
Male	99 (25.3)	39 (34.2)		
Female	292 (74.7)	75 (65.8)		
Tumor size (mm)			23.161	**<0.001**
≤ 4	260 (66.7)	47 (41.6)		
> 4	130 (33.3)	66 (58.4)		
Lymph node metastasis			17.891	**<0.001**
No	192 (55.3)	35 (32.1)		
Yes	155 (44.7)	74 (67.9)		
Distant metastasis			3.189	0.074
No	213 (54.6)	73 (64.0)		
Yes	177 (45.4)	41 (36.0)		
Stage			22.880	**<0.001**
I–II	281 (72.2)	55 (48.2)		
III–IV	108 (27.8)	59 (51.8)		

CTHRC1: Collagen triple helix repeat containing 1.

**Table 6:** Association of CTHRC1 expression with clinicopathological parameters in colon cancer patients based on clinical tissues

**Characteristics**	**Low expression of CTHRC1 (%)**	**High expression of CTHRC1 (%)**	***χ^2^***	***P***
*n*	25	25		
Age			0.368	0.544
≤ 55	7 (28.0)	9 (36.0)		
> 55	18 (72.0)	16 (64.0)		
Gender			0.058	0.771
Male	16 (64.0)	15 (60.0)		
Female	9 (36.0)	10 (40.0)		
Tumor infiltration depth			4.504	**0.034**
≤ T2	12 (48.0)	4 (16.0)		
T3-T4	13 (52.0)	21 (84.0)		
Lymph node metastasis			0.347	0.556
No	17 (68.0)	15 (60.0)		
Yes	8 (32.0)	10 (40.0)		
Distant metastasis			0.149	0.700
No	22 (88.0)	20 (80.0)		
Yes	3 (12.0)	5 (20.0)		
Stage			4.504	**0.034**
I	12 (48.0)	4 (16.0)		
II–IV	13 (52.0)	21 (84.0)		

CTHRC1: Collagen triple helix repeat containing 1.

**Table 7:** Association of CTHRC1 expression with clinicopathological parameters in thyroid cancer patients based on clinical tissues

**Characteristics**	**Low expression of CTHRC1 (%)**	**High expression of CTHRC1 (%)**	***χ^2^***	***P***
*n*	25	25		
Age			0.081	0.777
≤ 45	11 (44.0)	12 (48.0)		
> 45	14 (56.0)	13 (52.0)		
Gender			1.389	0.239
Male	7 (28.0)	11 (44.0)		
Female	18 (72.0)	14 (56.0)		
Tumor size (cm)			5.556	**0.018**
< 4	20 (80.0)	12 (48.0)		
≥ 4	5 (20.0)	13 (52.0)		
Lymph node metastasis			4.023	**0.045**
No	14 (56.0)	7 (28.0)		
Yes	11 (44.0)	18 (72.0)		
Distant metastasis			0.125	0.724
No	21 (84.0)	19 (76.0)		
Yes	4 (16.0)	6 (24.0)		
Stage			1.282	0.258
I–II	15 (60.0)	11 (44.0)		
III–IV	10 (40.0)	14 (56.0)		

CTHRC1: Collagen triple helix repeat containing 1.

To analyze the diagnostic value of CTHRC1 in colon cancer, thyroid cancer, and melanoma patients, we performed an ROC curve analysis. As shown in Figure 3D–3F, the AUC was 0.977 for the diagnosis of colon cancer, 0.654 for the diagnosis of thyroid cancer, and 0.596 for the diagnosis of melanoma.

Therefore, the results of the survival analysis showed that CTHRC1 expression was significantly associated with survival in patients with colon cancer, thyroid cancer, and melanoma. The ROC curves indicated that CTHRC1 is an important risk factor for adjuvant diagnosis of these three tumors, with the highest diagnostic value in colon cancer.

### Association of CTHRC1 expression with clinicopathological features in colon cancer, thyroid cancer, and melanoma patients

Next, the association of CTHRC1 expression with clinicopathological parameters in colon cancer, thyroid cancer, and melanoma patients (including age, gender, tumor size, tumor infiltration depth, lymph node metastasis, distant metastasis, and clinical stage) was analyzed based on the TCGA data. CTHRC1 expression levels were categorized into high and low groups using the “sur.cut” value calculated by the “survminer” package as a cutoff classification. In colon cancer patients, expression of CTHRC1 was positively correlated with tumor infiltration depth (*P* ═ 0.019) and clinical stage (*P* ═ 0.034), while no significant correlation was found between CTHRC1 expression and patients’ age, gender, lymph node metastasis, or distant metastasis (*P* > 0.05) (Table 4). Expression of CTHRC1 was positively correlated with patient tumor size (*P* < 0.001), lymph node metastasis (*P* < 0.001), and clinical stage (*P* < 0.001), and there was no significant correlation between CTHRC1 expression and patients’ age, gender, or distant metastasis (*P* > 0.05) in patients with thyroid cancer (Table 5). In melanoma patients, CTHRC1 expression was correlated with patient age (*P* ═ 0.026), but no significant correlation was found between CTHRC1 expression and patients’ gender, tumor size, lymph node metastasis, distant metastasis, or clinical stage (*P* > 0.05) (Table S1).

Furthermore, we collected 50 colon cancer tissues, 50 thyroid cancer tissues, and 50 melanoma tissues for clinical analysis from the Department of Pathology, First Affiliated Hospital of Anhui Medical University, and the RNA levels of CTHRC1 were examined by qRT-PCR. The association of CTHRC1 expression with clinicopathological parameters was also studied. In colon cancer patients, expression of CTHRC1 was positively correlated with tumor infiltration depth (*P* ═ 0.034) and clinical stage (*P* ═ 0.034), but no significant correlation was found with patients’ age, gender, lymph node metastasis, or distant metastasis (*P*>0.05) (Table 6). In thyroid cancer patients, CTHRC1 expression was positively correlated with tumor size (*P* ═ 0.018) and lymph node metastasis (*P* ═ 0.045), but there was no significant correlation between CTHRC1 expression and patients’ age, gender, distant metastasis, or clinical stage (*P* > 0.05) (Table 7). In melanoma patients, CTHRC1 expression was not significantly associated with patients’ age, gender, tumor size, lymph node metastasis, distant metastasis, or clinical stage (*P* > 0.05) (Table S2).

Collectively, these data suggest that CTHRC1 is associated with poor prognosis in human colon cancer and thyroid cancer, but the association between CTHRC1 and patient prognosis was not significant in human melanoma.

### Molecular interactions and signaling pathway analysis of CTHRC1

**Figure 4.**
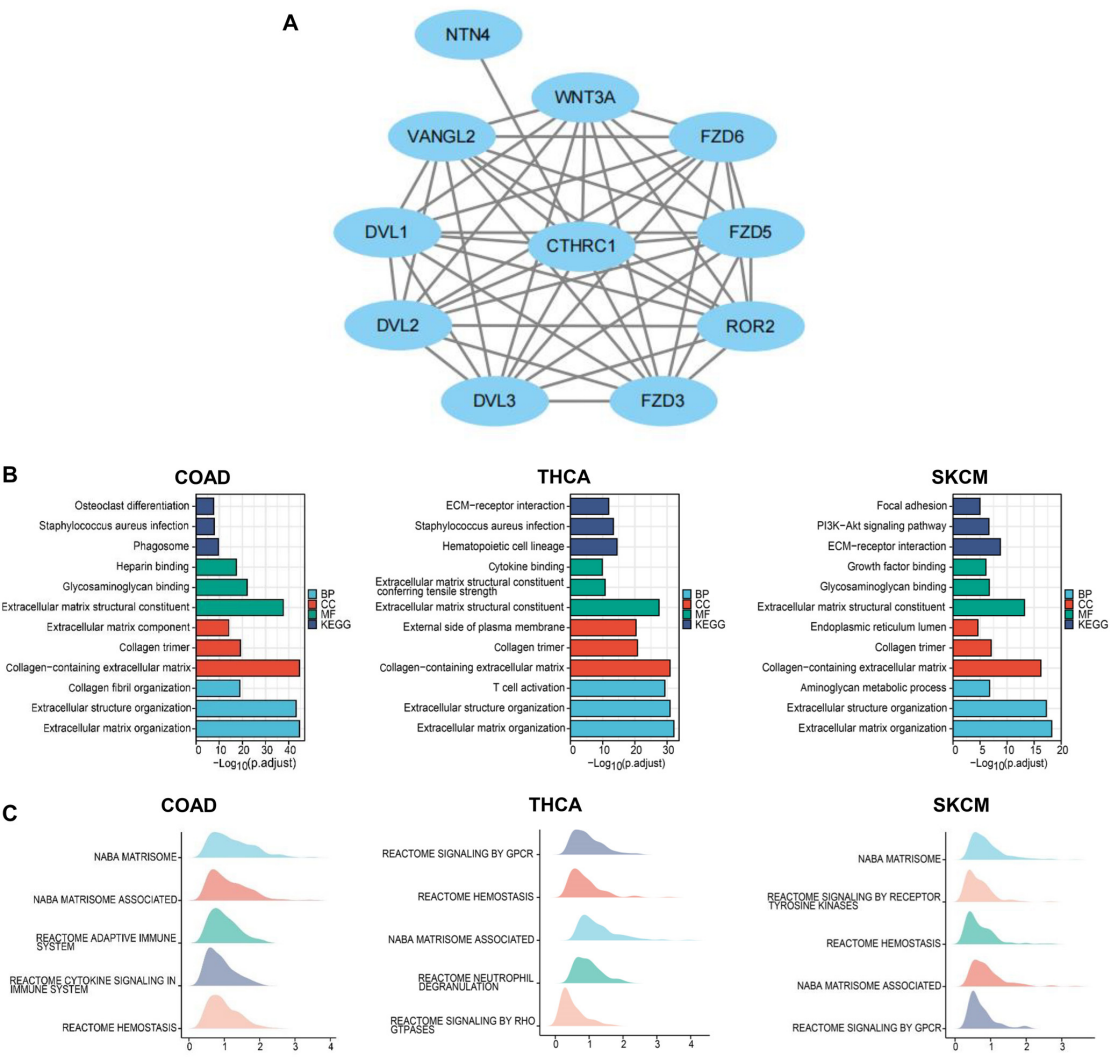
**PPI and molecular signaling pathway enrichment analyses of CTHRC1 in colon cancer, thyroid cancer, and melanoma.** (A) PPI network of CTHRC1 analyzed using the STRING tool; (B) Histogram showing the GO and KEGG pathway analyses of CTHRC1; (C) Top5 GSEA (gene set enrichment analysis) results for CTHRC1. CTHRC1: Collagen triple helix repeat containing 1; PPI: Protein–protein interaction; GO: Gene ontology; KEGG: Kyoto Encyclopedia of Genes and Genomes.

Based on the data from TCGA, molecular interactions and signaling pathways related to CTHRC1 were analyzed. As shown in Figure 4A, the potential co-expression genes of CTHRC1 were identified using the STRING tool and CTHRC1, DVL1, DVL2, DVL3, FZD3, ROR2, FZD5, FZD6, WNT3A, NTN4, and VANGL2 formed a network in human cancers.

Moreover, enrichment analysis showed that CTHRC1 was associated with signaling pathways including osteoclast differentiation, *Staphylococcus aureus* infection, phagosome, heparin binding, glycosaminoglycan binding, extracellular matrix structural constituent, extracellular matrix component, collagen trimer, collagen-containing extracellular matrix, collagen fibril organization, extracellular structure organization, and extracellular matrix organization in colon cancer. In thyroid cancer, CTHRC1 was associated with signaling pathways including ECM-receptor interaction, *S. aureus* infection, hematopoietic cell lineage, cytokine binding, extracellular matrix structural constituent conferring tensile strength, extracellular matrix structural constituent, external side of the plasma membrane, collagen trimer, collagen-containing extracellular matrix, T cell activation, extracellular structure organization, and extracellular matrix organization. In melanoma, CTHRC1 was associated with signaling pathways including focal adhesion, PI3K-Akt signaling pathway, ECM–receptor interaction, growth factor binding, glycosaminoglycan binding, extracellular matrix structural constituent, endoplasmic reticulum lumen, collagen trimer, collagen-containing extracellular matrix, aminoglycan metabolic process, extracellular structure organization, and extracellular matrix organization (Figure 4B).

Using GSEA analysis, NABA matrisome, NABA matrisome-associated, reactome adaptive immune system, reactome cytokine signaling in the immune system, and reactome hemostasis were found to be associated with CTHRC1 in colon cancer; reactome signaling by GPCR, reactome hemostasis, NABA matrisome-associated, reactome neutrophil degranulation, and reactome signaling by Rho-GTPases were associated with CTHRC1 in thyroid cancer; associations were observed between NABA matrisome, reactome signaling by receptor tyrosine kinases, reactome hemostasis, NABA matrisome-associated, and reactome signaling by GPCR and CTHRC1 in melanoma (Figure 4C).

### Association of CTHRC1 expression with immune cell infiltration and immunotherapy markers in colon cancer, thyroid cancer, and melanoma

**Figure 5.**
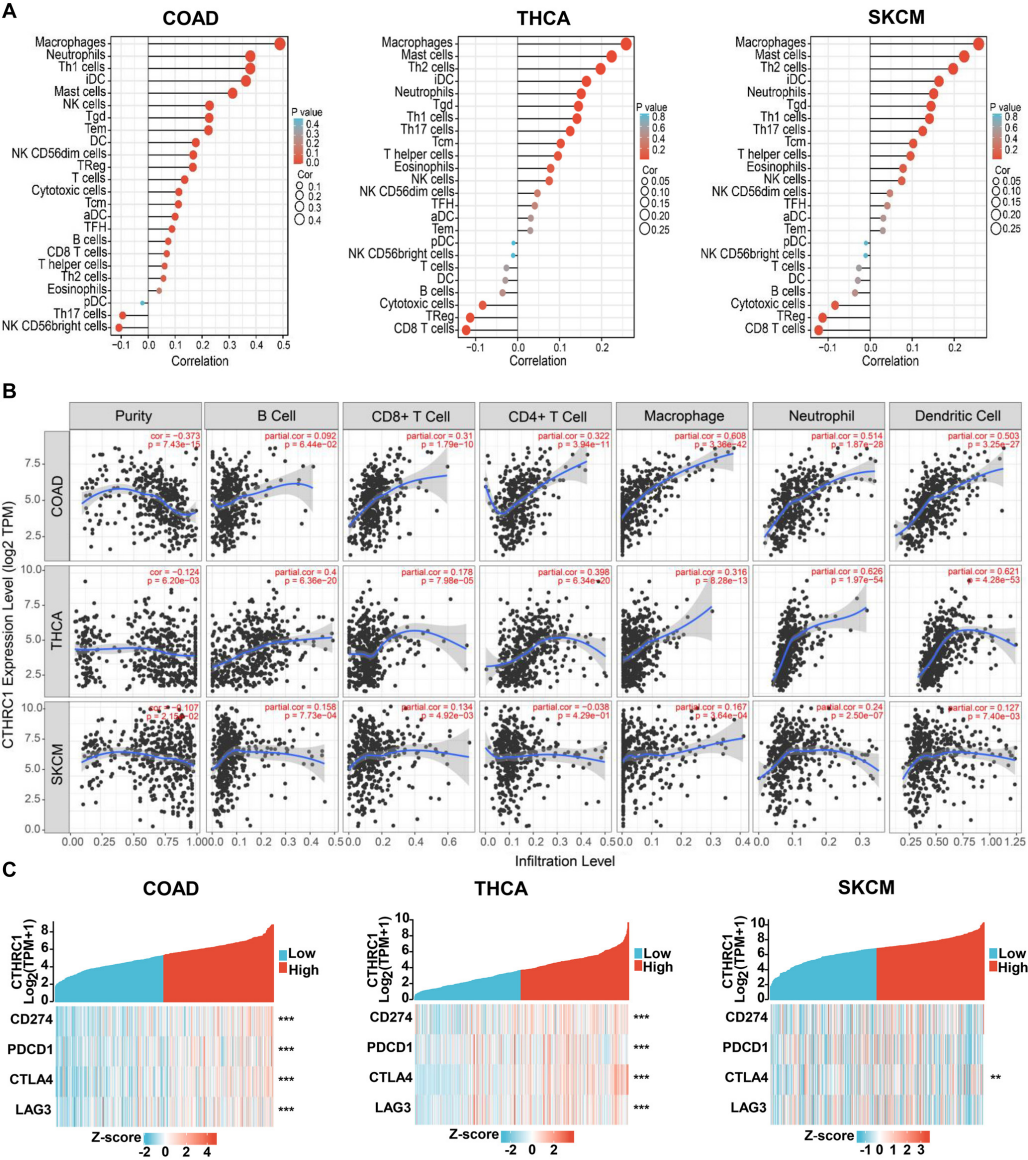
**Correlation analysis of CTHRC1 expression with immune cell infiltration and immunotherapy markers in colon cancer, thyroid cancer, and melanoma.** (A) Correlation of CTHRC1 expression with 24 types of immune cells calculated using Cibersort; (B) Correlation of CTHRC1 expression with respective immune cells based on the TIMER database; (C) Heatmap of CTHRC1 associated immunotherapy markers CD274, PDCD1, CTLA4 and LAG3. **P < 0.01; ***P < 0.001. CTHRC1: Collagen triple helix repeat containing 1.

Furthermore, we analyzed the correlation between CTHRC1 expression and immune cell infiltration in colon cancer, thyroid cancer, and melanoma. In colon cancer, many types of immune cell infiltration (especially macrophages, neutrophils, dendritic cells, CD4+ T cells, and CD8+ T cells) were positively correlated with CTHRC1 expression, while a few types of immune cells, including Th17 cells and NK CD56 bright cells, were negatively correlated with CTHRC1 expression (Figure 5A and 5B). In thyroid cancer, immune cells (especially neutrophils, dendritic cells, B cells, CD4+ T cells, and macrophages) were also positively correlated with CTHRC1 expression, while Th17 cell infiltration was negatively correlated with CTHRC1 expression (Figure 5A and 5B). In melanoma, immune cell infiltration, such as neutrophils and macrophages, was positively correlated with CTHRC1 expression, but the correlation of CTHRC1 expression with immune cell infiltration was not as significant as in colon cancer and thyroid cancer (respective correlation coefficients were smaller) (Figure 5A and 5B).

Moreover, we examined the correlation between CTHRC1 expression and immunotherapy markers, including CD274, PDCD1, CTLA4, and LAG3 in colon cancer, thyroid cancer, and melanoma. As shown in Figure 5C, CD274, PDCD1, CTLA4, and LAG3 were significantly positively correlated with CTHRC1 expression in colon cancer and thyroid cancer; while only CTLA4 was significantly positively correlated with CTHRC1 expression in melanoma.

Therefore, our data indicated that CTHRC1 expression was significantly correlated with multiple types of immune cell infiltration and immunotherapy marker expression in colon cancer and thyroid cancer, but not as significantly in melanoma. CTHRC1 might play an important role in immunotherapy for colon cancer and thyroid cancer.

### Patients with BRAF(V600E) mutation showed elevated CTHRC1 expression and immune cell infiltration in clinical tissues of colon cancer and thyroid cancer

**Figure 6.**
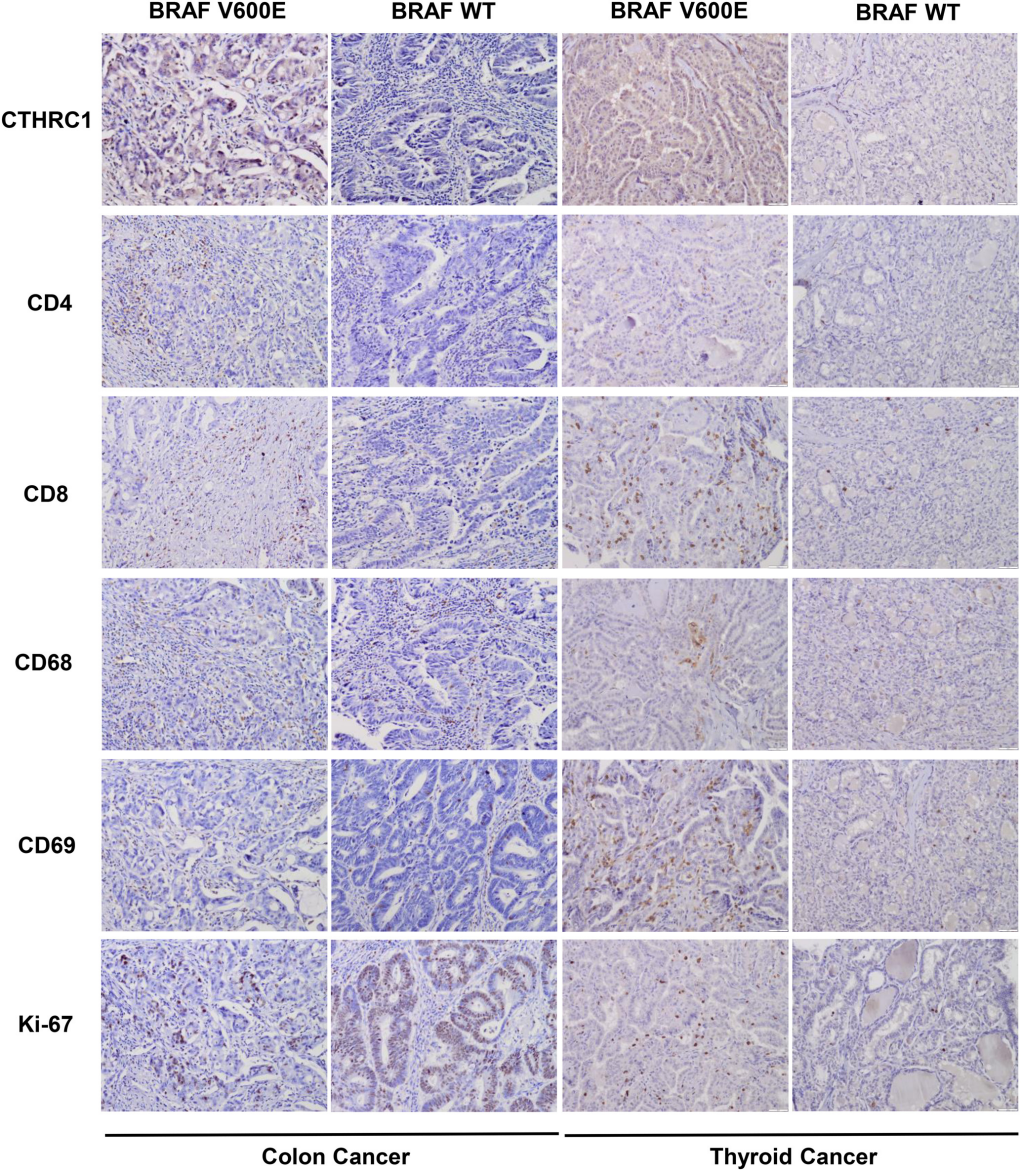
**CTHRC1 and immune cell marker levels in colon cancer and thyroid cancer clinical tissues with/without BRAF(V600E) mutation.** IHC showing CTHRC1, CD4, CD8, CD68, CD69, and Ki-67 protein levels in colon cancer and thyroid cancer clinical tissues with/without BRAF(V600E) mutation. Representative photographs are shown. BRAF(V600E), BRAF(V600E) mutation; BRAF WT, BRAF wild-type. CTHRC1: Collagen triple helix repeat containing 1; IHC: Immunohistochemistry.

**Figure S1.**
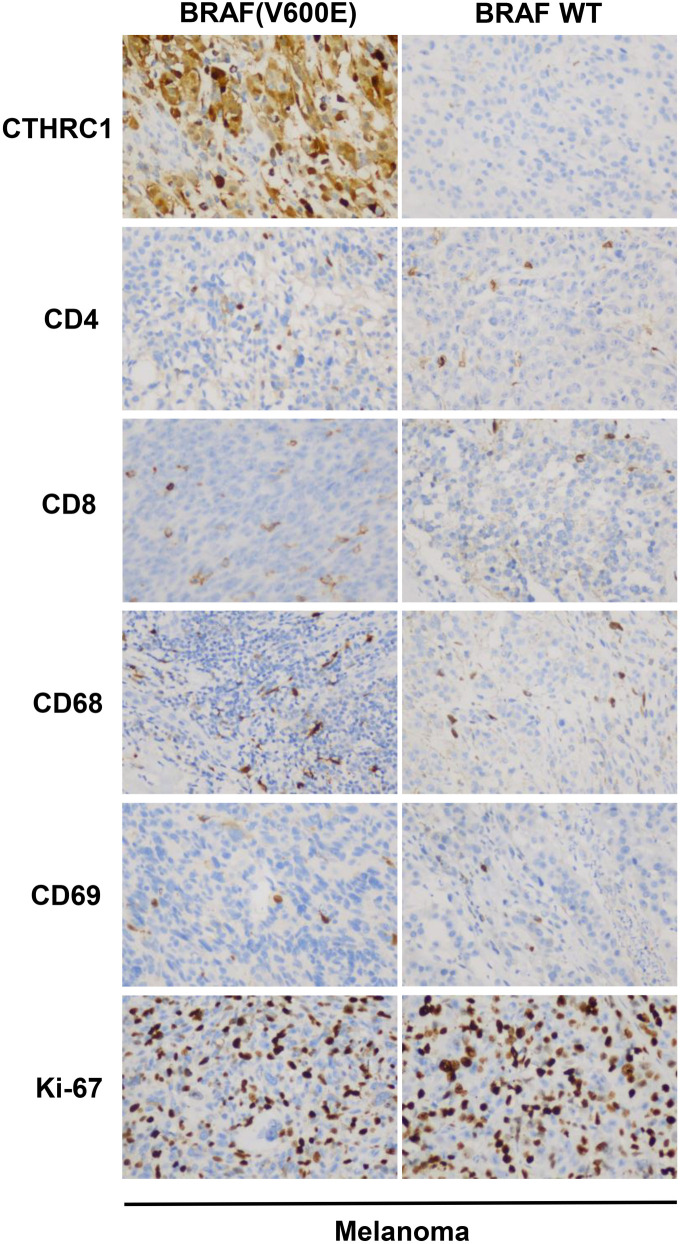
**CTHRC1 and immune cell marker levels in melanoma tissues with/without BRAF(V600E) mutation.** IHC to show CTHRC1, CD4, CD8, CD68, CD69, and Ki-67 protein levels in melanoma tissues with/without BRAF(V600E) mutation. Representative photographs were showed. BRAF(V600E), BRAF(V600E) mutation, BRAF WT, BRAF wild-type. CTHRC1: Collagen triple helix repeat containing 1; IHC: Immunohistochemistry.

For further study, we collected 15 colon cancer tissues with the BRAF(V600E) mutation, 15 wild-type BRAF colon cancer tissues (WT), 15 thyroid cancer tissues with the BRAF(V600E) mutation, 15 wild-type BRAF thyroid cancer tissues (WT), 15 melanoma tissues with the BRAF(V600E) mutation, and 15 wild-type BRAF melanoma tissues (WT). We examined the protein levels of CTHRC1, immune cell markers, such as CD4, CD8, CD68, and CD69, and the proliferation marker Ki-67 by IHC. As shown in Figure 6 and Table 8, protein levels of CTHRC1 were significantly higher in BRAF(V600E) mutant tissues compared with wild-type tissues (*P* ═ 0.021), and CD4-positive cells (*P* ═ 0.027), CD8-positive cells (*P* ═ 0.003), CD69 positive cells (neutrophils) (*P* ═ 0.025) were more enriched in BRAF(V600E) mutant tissues compared with wild-type tissues. However, there were no significant differences in CD68-positive cells (macrophages) or Ki-67-positive cells between BRAF(V600E) mutant colon cancer tissues and wild-type colon cancer tissues (*P* > 0.05) in colon cancer. In thyroid cancer, protein levels of CTHRC1 were also significantly higher in BRAF(V600E) mutant tissues compared with wild-type tissues (*P* ═ 0.009), and CD4-positive cells (*P* ═ 0.021), CD8-positive cells (*P* ═ 0.025), and CD69-positive cells (neutrophils) (*P* ═ 0.025) were more enriched in BRAF(V600E) mutant tissues compared with wild-type tissues. However, there were no significant differences in CD68-positive cells (macrophages) or Ki-67-positive cells between BRAF(V600E) mutant thyroid cancer tissues and wild-type thyroid cancer tissues (*P* > 0.05), as shown in Figure 6 and Table 9. However, as demonstrated in Figure S1 and Table S3, the protein levels of CTHRC1, immune cell markers, such as CD4, CD8, CD68, and CD69, and the proliferation marker Ki-67 were not significantly different between BRAF(V600E) mutant melanoma tissues and wild-type melanoma tissues (*P* > 0.05).

Therefore, the BRAF(V600E) mutation was associated with CTHRC1 expression, and both were correlated with immune cell infiltration including CD4+ T cells, CD8+ T cells, and neutrophils, in colon cancer and thyroid cancer patients.

### High level of CTHRC1 correlated with decreased sensitivity to BRAF(V600E) mutation-targeted drugs

**Figure 7.**
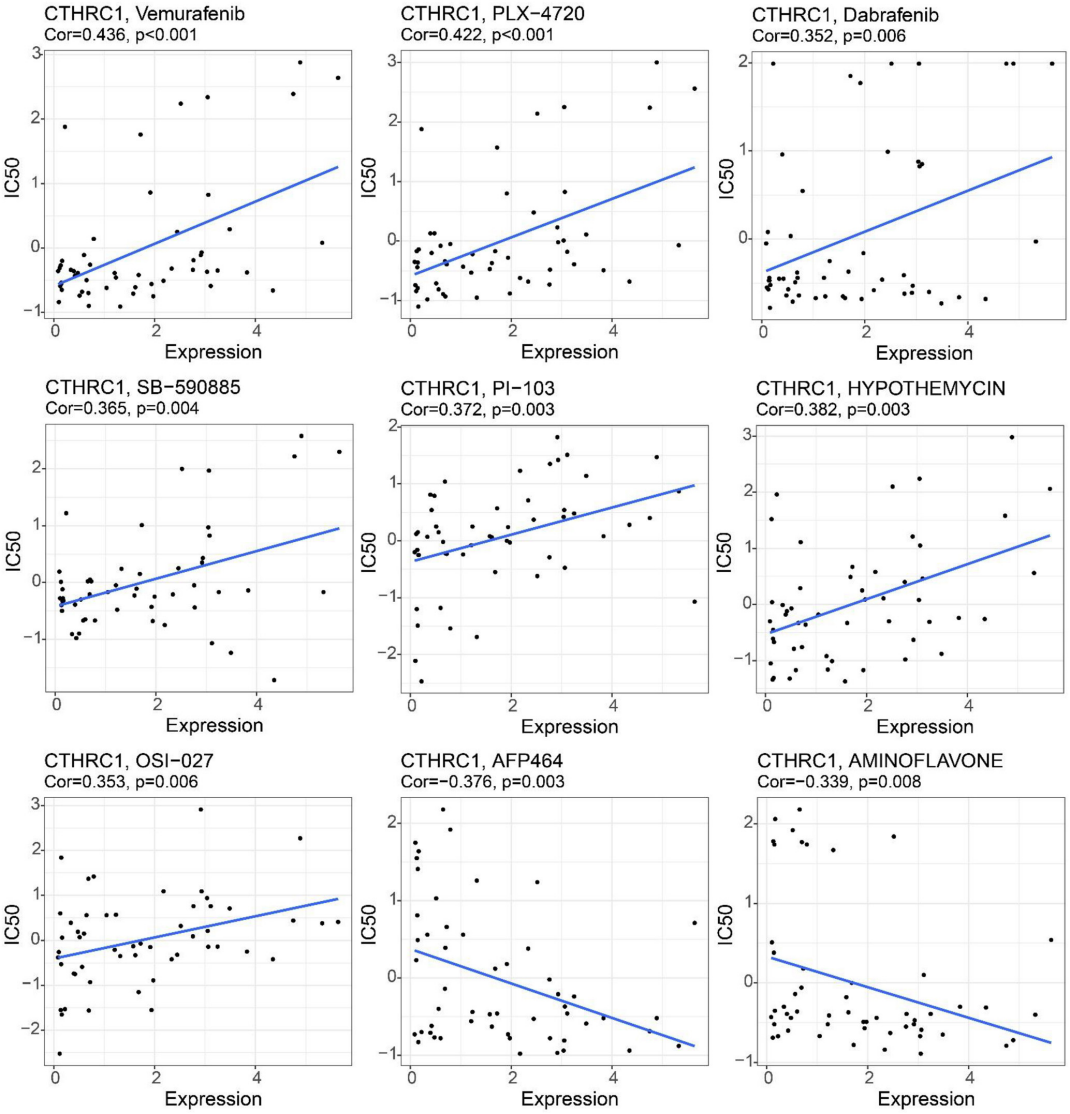
**Correlation analysis of CTHRC1 expression with anticancer drug sensitivity.** The correlations of CTHRC1 expression with anticancer drug sensitivity (Vemurafenib, PLX-4720, Dabrafenib, SB-590885, PI-103, Hypothemycin, OSI-027, AFP464, Aminoflavone) were analyzed based on the CellMiner database. CTHRC1: Collagen triple helix repeat containing 1.

Based on database analysis, we examined the correlation of CTHRC1 expression with the response to anti-tumor drugs. Vemurafenib, PLX-4720, Dabrafenib, and SB-590885 are four commonly used targeted drugs for BRAF(V600E) mutant tumors. As shown in Figure 7, the IC50 values of Vemurafenib, PLX-4720, Dabrafenib, and SB-590885 were all significantly positively correlated with CTHRC1 expression (Vemurafenib, cor ═ 0.436, PLX-4720, cor ═ 0.422, Dabrafenib, cor ═ 0.352, SB-590885, cor ═ 0.365, all *P* < 0.01). Therefore, high CTHRC1 expression levels might be correlated with decreased sensitivity to BRAF(V600E) mutation-targeted drugs.

Moreover, analysis of other drugs showed that the IC50 values of PI-103, Hypothemycin, and OSI-027 were also positively correlated with CTHRC1 expression in human cancers, while IC50 values of AFP464 and Aminoflavone showed negative correlations with CTHRC1 expression in human cancers. These data suggested that CTHRC1 is an important biomarker for evaluating anti-tumor drug sensitivity.

## Discussion

In this study, we demonstrated that CTHRC1 was positively correlated with the BRAF(V600E) mutation in human colon cancer, thyroid cancer, and melanoma. In colon cancer and thyroid cancer patients, CTHRC1 expression levels were negatively associated with OS, and univariate COX regression analysis showed that CTHRC1 was an important risk factor correlated with patient OS rates. Expression levels of CTHRC1 were significantly correlated with tumor infiltration depth and clinical stage in colon cancer patients and with tumor size, lymph node metastasis, and clinical stage in thyroid cancer patients. These results suggest that CTHRC1 is an oncogenic biomarker in colon cancer and thyroid cancer. As reported in previous studies, CTHRC1 promotes human colorectal cancer cell proliferation and invasiveness by activating the Wnt/PCP signaling pathway [26]; CTHRC1 promotes liver metastasis of colorectal cancer through the TGF-β pathway [37]. Additionally, Pang et al. [38] demonstrated that CTHRC1 is a potential diagnostic and prognostic indicator of colon adenocarcinoma, and overexpression of CTHRC1 was associated with poor prognosis in colorectal cancer patients [39]. Recent studies have also reported that CTHRC1 is associated with the onset and malignant transformation of papillary thyroid carcinoma [27]. Furthermore, CTHRC1 has been shown to promote cell proliferation and inhibit apoptosis by activating the ERK1/2 signaling pathway in papillary thyroid carcinoma [40]. These findings are consistent with our current results. We provide compelling evidence that CTHRC1 is linked to the BRAF(V600E) mutation in both colon and thyroid cancers, further highlighting the significant role of CTHRC1 in influencing patient prognosis. On the other hand, although CTHRC1 was positively correlated with the BRAF(V600E) mutation in melanoma, CTHRC1 expression levels were positively associated with the OS rate in melanoma patients, which was the opposite with colon cancer and thyroid cancer. Additionally, there was no significant correlation between CTHRC1 expression and patients’ gender, tumor size, lymph node metastasis, distant metastasis, or clinical stage in melanoma. Despite the use of BRAF(V600E) targeted drugs in melanoma [41], the BRAF(V600E) mutation is actually more frequent in melanocytic nevi compared with melanoma [42]. Damsky et al. [43] reported that the BRAF(V600E) mutation induces mole formation in mice but rarely leads to melanoma formation. Therefore, while CTHRC1 and the BRAF(V600E) mutation serve as diagnostic and prognostic biomarkers in melanoma, they cannot simply be considered oncogenic biomarkers and exhibit tissue specificity.

**Table 8:** Protein levels of respective markers in BRAF(V6000E) mutant (Mut) and wild type (WT) colon cancer tissues examined by IHC

**Group**	***n***	**CTHRC1 expression**		**CD4 expression**		**CD8 expression**		**CD68 expression**		**CD69 expression**		**Ki-67 expression**	
		**Low**	**High**	**Low**	**High**	**Low**	**High**	**Low**	**High**	**Low**	**High**	**Low**	**High**
WT	15	9	6	11	4	11	4	7	8	12	3	6	9
Mut	15	2	13	4	11	2	13	6	9	5	10	7	8
*P*		**0.021** ^†^		**0.027** ^†^		**0.003** ^†^		1.000^†^		**0.025** ^†^		1.000^†^	

^†^for MWU test. IHC: Immunohistochemistry.

**Table 9:** Protein levels of respective markers in BRAF(V6000E) mutant (Mut) and wild type (WT) thyroid cancer tissues examined by IHC

**Group**	*n*	**CTHRC1 expression**		**CD4 expression**		**CD8 expression**		**CD68 expression**		**CD69 expression**		**Ki-67 expression**	
		**Low**	**High**	**Low**	**High**	**Low**	**High**	**Low**	**High**	**Low**	**High**	**Low**	**High**
WT	15	11	4	13	2	12	3	11	4	12	3	11	4
Mut	15	3	12	4	11	5	10	8	7	5	10	6	9
*P*		**0.009** ^†^		**0.021** ^†^		**0.025** ^†^		0.450^†^		**0.025** ^†^		0.139^†^	

^†^for MWU test. IHC: Immunohistochemistry.

In the molecular network study, we found that DVL1, DVL2, DVL3, FZD3, ROR2, FZD5, FZD6, WNT3A, NTN4, and VANGL2 were involved in the CTHRC1 pathway. Remarkably, three members of the FZD protein family (frizzled transmembrane receptor family): FZD3, FZD5, and FZD6, were associated with CTHRC1. As previously reported, FZD3 is oncogenic in melanoma; high expression of FZD3 is correlated with poor OS in melanoma patients [44]. FZD5 was reported to be regulated through the Wnt signaling pathway, affecting Paneth cell differentiation and playing a promoting role in tumor development [45–47]. In Luo et al.’s study [48], FZD6 was identified as a potential biomarker for anaplastic thyroid cancer; FZD6 was also correlated with VEGFA expression, promoting vascularization and primary tumor spread in uveal melanoma [49]. Therefore, the CTHRC1/FZDs pathway may play important roles in colon cancer, thyroid cancer, and melanoma. Moreover, the network member WNT3A was involved in the induction of transcriptional co-activator YAP/TAZ, promoting the Wnt-FZD signaling axis, and acting as a key role in gene expression, osteogenic differentiation, and cell migration [50]. NTN4 was reported to enhance non-medullary thyroid cancer susceptibility [51], promote melanoma cell invasion [52], but inhibit primary and metastatic colorectal tumor progression [53]. ROR2, VANGL2, and DVLs were demonstrated to be involved in the WNT pathway [54, 55], serving as pivotal regulators of proliferation, metastasis, and development of colon cancer, thyroid cancer, and melanoma [56–59]. Therefore, the CTHRC1 network may play crucial roles in human colon cancer, thyroid cancer, and melanoma. However, the deeper mechanisms and functions of the CTHRC1 network in these cancers require further study.

Moreover, we found that many types of immune cell infiltration and immunotherapy markers were positively correlated with CTHRC1 expression in colon cancer, thyroid cancer, and melanoma (macrophages, neutrophils, dendritic cells, CD4+ T cells, CD8+ T cells/CD274, PDCD1, CTLA4, LAG3 in colon cancer; neutrophils, dendritic cells, B cells, CD4+ T cells, macrophages/CD274, PDCD1, CTLA4, LAG3 in thyroid cancer; neutrophils, macrophages/CTLA4 in melanoma). As previously reported, CD274 and PDCD1 are widely recognized biomarkers for predicting the efficacy of PD-1/PD-L1 immune checkpoint-based immunotherapy in human tumors [60]. Additionally, the phagocytic ability of tumor-associated macrophages to tumor cells was negatively correlated with PDCD1 expression in colon cancer [61]. Metastatic melanoma secretes CD274-loaded extracellular vesicles to inhibit the function of CD8+ T cells and promote tumor growth [62]. In a previous study, expression of CTLA4 was demonstrated to suppress immune cell activation in colon cancer [63], positively correlated with multiple immune cell infiltrations in papillary thyroid carcinoma [64], and potentially regulated lymph node T cell proliferation in the early immune response of melanoma [65]. LAG3 is another biomarker for immunotherapy, and antagonists of LAG3 enhance the antitumor efficacy of PD-L1 blockade therapy in colon cancer and melanoma [66, 67]. Based on the evidence we have provided, CTHRC1 expression was significantly associated with immune cell infiltration and immunotherapy markers. We can conclude that CTHRC1 plays a crucial role in the immune response in colon cancer, thyroid cancer, and melanoma, and CTHRC1 could be used as a potential target for immunotherapy.

BRAF(V600E)-targeted drugs (including Vemurafenib, PLX-4720, Dabrafenib, and SB-590885) have been effectively used for the treatment of colon cancer, thyroid cancer, and melanoma with the BRAF(V600E) mutation [68–71]. However, drug resistance has made it difficult for patients to achieve the desired therapeutic effect [72–75]. The mechanisms involved in BRAF(V600E)-targeted drug resistance are complex. It has been reported that NRG-1β activates ErbB-3 to promote Vemurafenib resistance in BRAF(V600E) colon cancer stem cells (CSCs) [76]. Overexpression of HMGB1 has been shown to reduce the sensitivity of BRAF(V600E) mutant thyroid cancer cells to Vemurafenib by increasing cell viability and decreasing apoptosis and caspase-3 activity [73]. RAC.amplification has also been reported to be associated with Dabrafenib resistance in papillary thyroid carcinoma [77]. In this study, we provided evidence that the IC50 values of Vemurafenib, PLX-4720, Dabrafenib, and SB-590885 were all significantly positively correlated with CTHRC1 expression levels. Therefore, high expression of CTHRC1 was correlated with resistance to BRAF(V600E)-targeted drugs. CTHRC1 inhibitors could potentially be used as adjuvant treatment alongside BRAF(V600E)-targeted drugs in BRAF(V600E) mutant colon cancer, thyroid cancer, and melanoma.

## Conclusion

In summary, this study demonstrated that CTHRC1 was correlated with BRAF(V600E), prognosis, and clinicopathological features in colon cancer, thyroid cancer, and melanoma. CTHRC1 expression was also associated with immune cell infiltration, immunotherapy markers, and resistance to BRAF(V600E) inhibitors. Therefore, CTHRC1 could be used as a diagnostic, prognostic, and adjuvant therapeutic biomarker in human colon cancer, thyroid cancer, and melanoma.

## Supplemental data

**Table S1:** Association of CTHRC1 expression with clinicopathological features in melanoma patients based on TCGA database

**Characteristics**	**Low expression of CTHRC1 (%)**	**High expression of CTHRC1 (%)**	***χ^2^***	***P***
*n*	286	185		
Age			4.951	**0.026**
≤ 55	108 (38.4)	89 (48.9)		
> 55	173 (61.6)	93 (51.1)		
Gender			1.692	0.193
Male	184 (64.3)	108 (58.4)		
Female	102 (35.6)	77 (41.6)		
Tumor size (mm)			2.549	0.110
< 2	85 (35.1)	66 (43.1)		
≥ 2	157 (64.9)	87 (56.9)		
Lymph node metastasis			0.150	0.699
No	140 (56.0)	95 (57.9)		
Yes	110 (44.0)	69 (42.1)		
Distant metastasis			0.800	0.371
No	255 (95.1)	163 (93.1)		
Yes	13 (4.9)	12 (6.9)		
Stage			0.071	0.790
I–II	147 (55.5)	91 (54.2)		
III–IV	118 (44.5)	77 (45.8)		

CTHRC1: Collagen triple helix repeat containing 1.

**Table S2:** Association of CTHRC1 expression with clinicopathological parameters in melanoma patients based on clinical tissues

**Characteristics**	**Low expression of CTHRC1(%)**	**High expression of CTHRC1(%)**	***χ^2^***	***P***
*n*	25	25		
Age			0.739	0.390
≤ 55	9 (36.0)	12 (48.0)		
> 55	16 (64.0)	13 (52.0)		
Gender			0.085	0.771
Male	15 (60.0)	16 (64.0)		
Female	10 (40.0)	9 (36.0)		
Tumor size (cm)			0.764	0.382
< 2	8 (32.0)	11 (44.0)		
≥ 2	17 (68.0)	14 (56.0)		
Lymph node metastasis			0.439	0.508
No	18 (72.0)	20 (80.0)		
Yes	7 (28.0)	5 (20.0)		
Distant metastasis			0.466	0.495
No	21 (84.0)	18 (72.0)		
Yes	4 (16.0)	7 (18.0)		
Stage			0.325	0.569
I–II	13 (52.0)	15 (60.0)		
III–IV	12 (48.0)	10 (40.0)		

CTHRC1: Collagen triple helix repeat containing 1.

**Table S3:** Protein levels of respective markers in BRAF(V6000E) mutant (Mut) and wild type (WT) melanoma tissues examined by immunohistochemistry

**Group**	***n***	**CTHRC1 expression**		**CD4 expression**		**CD8 expression**		**CD68 expression**		**CD69 expression**		**Ki-67 expression**	
		**low**	**high**	**low**	**high**	**low**	**high**	**low**	**high**	**low**	**high**	**low**	**high**
WT	15	10	5	9	6	10	5	10	5	8	7	5	10
Mut	15	5	10	8	7	8	7	9	6	6	9	4	11
*P*		0.143^†^		1.000^†^		0.710^†^		1.000^†^		0.715^†^		1.000^†^	

^†^For MWU test. CTHRC1: Collagen triple helix repeat containing 1.

## Data Availability

All data generated or analyzed during this study are included in this article. The original experimental data related to the research results in the article will be provided without reservation if necessary.
